# Risks of Novel Coronavirus Disease (COVID-19) in Pregnancy; a Narrative Review

**Published:** 2020-03-23

**Authors:** Latif Panahi, Marzieh Amiri, Somaye Pouy

**Affiliations:** 1 Master Student of Nursing, School of Nursing and Midwifery, Guilan University of Medical Sciences, Rasht, Iran.; 2 Department of Emergency Medicine, Razi Hospital, School of Medicine, Guilan University of Medical Sciences, Rasht, Iran.; 3 PhD Student of Nursing, School of Nursing and Midwifery, Guilan University of Medical Sciences, Rasht, Iran.

**Keywords:** COVID-19, pregnancy, infectious disease transmission, vertical, coronavirus, severe acute respiratory syndrome coronavirus 2

## Abstract

**Introduction:**

The outbreak of the new Coronavirus in China in December 2019 and subsequently in various countries around the world has raised concerns about the possibility of vertical transmission of the virus from mother to fetus. The present study aimed to review published literature in this regard.

**Methods:**

In this narrative review, were searched for all articles published in various databases including PubMed, Scopus, Embase, Science Direct, and Web of Science using MeSH-compliant keywords including COVID-19, Pregnancy, Vertical transmission, Coronavirus 2019, SARS-CoV-2 and 2019-nCoV from December 2019 to March 18, 2020 and reviewed them. All type of articles published about COVID-19 and vertical transmission in pregnancy were included.

**Results:**

A review of 13 final articles published in this area revealed that COVID-19 can cause fetal distress, miscarriage, respiratory distress and preterm delivery in pregnant women but does not infect newborns. There has been no report of vertical transmission in pregnancy, and it has been found that clinical symptoms of COVID-19 in pregnant women are not different from those of non-pregnant women.

**Conclusion:**

Overall, due to lack of appropriate data about the effect of COVID-19 on pregnancy, it is necessary to monitor suspected pregnant women before and after delivery. For confirmed cases both the mother and the newborn child should be followed up comprehensively.

## Introduction:

Coronaviruses are among the main human and animal pathogens (1). The COVID-19 epidemic began in China and quickly spread to other countries and became a major health problem (2). The disease was first spread in Wuhan, the capital of Hubei province, China, and the quickly spread to other countries around the world, including Iran (3-5). Since the first case of COVID-19 in Wuhan, China, up to March 19^th^, 234073 people in the world have been infected with COVID-19 and 9840 people have died because of COVID-19 infection (6-9). 

On January 30, 2020, the World Health Organization (WHO) labeled the outbreak as a Public Health Emergency of International Concern (PHEIC). On February 12, 2020, WHO named the disease caused by the novel coronavirus “Coronavirus Disease 2019” (COVID-19). A team of international experts, with a range of specializations, has tried to manage this outbreak (10, 11). Pneumonia caused by COVID-19 is a highly contagious and infectious disease declared a health emergency by the World Health Organization (11-13). 

The exact way of disease transmission has not yet been determined, but the researchers found that the virus spreads through respiratory droplets like the flu, and air precautions are very necessary given the lack of information in this area (14).

With the spread of the coronavirus, concerns have been raised about its intrauterine transmission from mother to fetus in pregnant women (2, 3, 15). Viral pneumonia is one of the leading causes of pregnancy deaths worldwide (16). Important questions raised due to the spread of COVID-19 include: Are the symptoms of pneumonia in pregnant women different from those of non-pregnant women? How likely are maternal and neonatal mortality? Does it cause pregnancy complications or premature birth? and How much COVID-19 is transmitted to the baby (3, 17)? Given the importance of the issue and the lack of sufficient evidence, the present study aimed to review the published evidence in this regard.

## Methods:

This study is a narrative review designed to collect published literature and articles on intrauterine transmission of COVID-19 from mother to fetus. In this review, we searched for all articles published in various databases including PubMed, Scopus, Embase, Science Direct and Web of Science using MeSH-compliant keywords including COVID-19, Pregnancy, vertical transmission, Coronavirus 2019, SARS-CoV-2 and 2019-nCoV from December 2019 to March 11 2020 and then reviewed them. All original research studies, letters to the editor, and reviews published on the impact of COVID-19 on fetal health and intrauterine transmission of COVID-19 were included. 

The title and abstract of all published articles were analyzed separately using specific keywords by two researchers, the relevant articles were collected, and their results were summarized and reported. 

## Results:

***Characteristics of included studies***

Searching the databases using specific keywords yielded 913 articles. After elimination of duplicates and review of abstracts and titles, 15 of articles were deemed relevant. 3 of the 15 articles were excluded due to being written in Chinese and finally 13 articles were included in the study. No original research on COVID-19 has been published so far, and only five studies were designed as case study or case series. Other published studies were in the form of correspondence, commentary or letters to the editor. Characteristics of included studies are presented in table 1. 

***Analysis of the reports***

A total of 37 pregnant mothers with COVID-19 and 38 newborns (two were twins) were studied. The age range of mothers was 23-40 years. Of these, 29 had cesarean delivery and 8 had normal delivery. Of the 37 pregnant mothers, 7 reported preterm labor at 30-33 weeks of age and the rest had no preterm labor and all had delivery in the third trimester (between 34 and 40 weeks’ gestation). Only one study reported that the neonate died after birth. Of the 37 mothers, 6 had preterm labor, 6 had premature rupture of the membrane, 2 had abnormal amniotic fluid, and 2 had abnormal umbilical cord. 

None of the mothers needed mechanical ventilation after delivery and only received antiviral, antibiotic, and oxygen therapy through the nasal catheter. Only one parturient woman required ICU admission and oxygen through the Venturi mask and her neonate was admitted to the NICU ward for monitoring. 

The most common infected mothers' symptoms were fever, cough, and chest pain. On admission, the lungs of all mothers were normal, but chest CT scan reported unilateral and bilateral infiltrations. Out of the 37 studied mothers, 2 had clinical manifestations of COVID-19 during delivery, 2 showed symptoms after delivery and the rest of them had symptoms of COVID-19 during hospitalization and prenatal delivery. 

Chest CT scan was performed for all of them and the most commonly reported finding was ground glass opacity (GGO) with progressive to consolidations. In 35 mothers, chest CT scan before and after delivery revealed no changes, in four postpartum women chest CT scan results had improved and in one patient it had exacerbated.

The most common laboratory finding was lymphocytopenia. No antiviral medications were given to mothers during pregnancy. All of the studied women gave birth to a healthy baby, with an Apgar score of 8-10. No amniotic fluid abnormality, cyanosis, asphyxia, abortion, or congenital abnormalities at birth were reported. 

Samples were taken from neonate’s throat, umbilical cord, amniotic fluid, stool, neonatal blood samples and breast milk of mother immediately after birth for screening of SARV-19 infection via SARS-CoV2 RT-PCR. Regarding neonatal outcomes, no information is available on teratogenicity and transmission of infection via placenta in the first, second and third trimesters of pregnancy, during normal vaginal delivery and through breast milk. 

**Table 1:** Characteristics of included studies

**Author**	**Year**	**Country**	**Title**	**Key Finding**
Chen et al. (12)	2020	China	Clinical characteristics and intrauterine vertical transmission potential of COVID-19 infection in nine pregnant women: a retrospective review of medical records	COVID-19 in pregnant woman can cause fetal distress but does not infect newborns.
Chua et al. (18)	2020	China	From the frontlines of COVID‐19–How prepared are we as obstetricians: a commentary	No evidence of intrauterine infection of COVID-19 caused by vertical transmission for fetus. Infected or suspect mothers should refrain from breastfeeding. All mothers infected with COVID-19 should be monitored carefully during pregnancy and after delivery.
Liu et al. (17)	2020	China	Pregnancy and Perinatal Outcomes of Women with COVID-19 Pneumonia: A Preliminary Analysis	Pregnancy and childbirth did not aggravate the course of symptoms or CT features of COVID-19 Pneumonia.
Liu et al. (16)	2020	China	Coronavirus Disease 2019 (COVID-19) During Pregnancy: A Case Series	No evidence to suggest the potential risk of intrauterine vertical transmission.
Lu et al. (19)	2020	China	Coronavirus disease (COVID‐19) and neonate: What neonatologist need to know	There is currently no evidence of trans-placental transmission of SARSCoV‐2 from the mother to the newborn.
Mardani et al. (3)	2020	Iran	A Controversial Debate: Vertical Transmission of COVID-19 in Pregnancy	Neonates born to women with suspected or confirmed COVID-19 infection should be isolated for at least two weeks after birth and not be breastfed. If 2019-nCoV infection is confirmed during pregnancy, both the mother and fetus should be followed up extensively.
Qiao et al. (2)	2020	China	What are the risks of COVID-19 infection in pregnant women?	There is not sufficient evidence about intrauterine vertical transmission.
Rasmussen et al. (4)	2020	USA	Coronavirus Disease 2019 (COVID-19) and Pregnancy: What obstetricians need to know	Fetal distress and preterm delivery were seen in some newborns. The babies of all pregnant women with COVID-19 were tested for SARS-CoV-2 after delivery and had negative results.
Wang et al. (15)	2020	China	A case of 2019 Novel Coronavirus in a pregnant woman with preterm delivery	There is no evidence of fetus distress or neonatal infection with COVID-19. COVID-19 in pregnancy can be mild to severe and result in preterm delivery.
Zhu et al. (20)	2020	China	Clinical analysis of 10 neonates born to mothers with 2019-nCoV pneumonia	Perinatal 2019-nCoV infection may have adverse effects on newborns, causing problems such as fetal distress, premature labor, respiratory distress, thrombocytopenia accompanied by abnormal liver function, and even death.
Liang et al. (20)	2020	China	Novel corona virus disease (COVID-19) in pregnancy: What clinical recommendations to follow?	There is no evidence for vertical transmission of COVID-19 in pregnant woman. All mothers with COVID-19 should be monitored carefully.
Faver et al. (21)	2020	China	2019-nCoV epidemic: what about pregnancies?	Infection with COVID-19 in pregnant women can have adverse effects including miscarriage, fetal growth restriction, and preterm birth or death of the mother.
Schwartz et al. (22)	2020	China	Potential Maternal and Infant Outcomes from Coronavirus 2019-nCoV (SARS-CoV-2) Infecting Pregnant Women: Lessons from SARS, MERS, and Other Human Coronavirus Infections	There is limited knowledge regarding coronavirus infections that occur during pregnancy. Previous experiences with coronavirus infections in pregnancy indicate that these agents are capable of causing adverse clinical outcomes.

Based on reported cases, all neonates with confirmed COVID-19 had been infected after birth via cough of mother or other relatives, or through the infected environment and had an average time of symptom manifestation between 5 to 17 days after birth. 

The most common symptoms of COVID-19 in the studied infants were tachypnea, milk regurgitation, vomiting, cough, fever, pneumothorax, liver disorders, thrombocytopenia, and pulmonary changes in chest CT scan. All infants born to mothers with COVID-19 were fed formula.

## Discussion:

Based on the findings of the present study, no original research has been carried out on the possibility of vertical transmission of COVID-19 from mother to the fetus and it is essential to carry out effective research in this area. According to results of studies, infected or suspected mothers should be carefully monitored before and after delivery. They should avoid breastfeeding until it is confirmed that they are not infected with COVID-19. Also, Mothers and their neonates should be taken care of in isolated rooms in order to prevent neonatal transmission. 

mothers with confirmed COVID-19 should be treated with antibiotics and antiviral drugs after childbirth (18). In this regard, a study by Chen et al. in China on 9 pregnant mothers with COVID-19 found that none of the newborns had postpartum complications such as COVID-19 infection and prematurity (12). This finding is in line with the results of a previous study on SARS-CoV-1 that was done by Wong and colleagues (15). 

However, acourding to some studies, infection with COVID-19 during pregnancy can cause complications for both the mother and the fetus; including preterm delivery, respiratory distress, fetal distress, coaglopathy accompanied by liver dysfunction and death of the mother. The newborn and the mother with confirmed COVID-19 should be isolated in different rooms and be screened very carefully (4, 7, 21, 23). Also, according to Chen et al., the clinical symptoms of COVID-19 in pregnant women were not significantly different from those of non-pregnant women, with common symptoms including chest pain, shortness of breath, fever and lethargy (12). Also, Liu et al. found that Pregnancy and childbirth did not aggravate the course of symptoms or CT features of COVID-19 Pneumonia (16). Overall, due to lack of evidence, scientists and researchers could not confirm vertical transmission of COVID-19 infection from placenta, during delivery and breast milk in the perinatal period (17). In some studies, evaluating both cesarean and normal vaginal delivery in mothers with COVID-19 showed that neither type of delivery affected their newborns and all of the studied newborns were negative for COVID-19 infection (12, 15, 16, 24). Previously published studies have demonstrated that being affected with SARS during perinatal period is associated with a high prevalence of harmful maternal and neonatal side effects including disseminated intravascular coagulopathy, abrupt abortion, preterm childbirth, intrauterine growth retard, neonatal intubation and need of newborn to be admitted to neonatal intensive care unit, and organ failure (17, 25). Generally, our review of literature showed that pregnant women infected with COVID-19 and their newborns had less problems than would be anticipated for those with SARS-CoV-1 infection. Although the findings should be interpreted with percaution because of the small sample size, the results are mostly in line with the findings by Zhu and colleagues (24) that was done on ten newborn who were born to mothers with COVID-19 pneumonia. Also, the clinical manifastations reported in pregnant women with positive COVID-19 are similar to those reported for non-pregnant women infected with COVID-19 and relatively good clinical outcomes have been reported for COVID-19 infection in pregnant women compared with SARS-CoV-1 infection (26, 27). More studies in this area are recommended.
